# Correcting Lab Misinterpretations of Variants of Unknown Significance: A Case Study of EMILIN1 Variants in an Autosomal Recessive Disorder

**DOI:** 10.7759/cureus.78706

**Published:** 2025-02-07

**Authors:** Yutaka Furuta, Rory J Tinker, Ryan M Dahlhauser, John A Phillips

**Affiliations:** 1 Medical Genetics, Vanderbilt University Medical Center, Nashville, USA

**Keywords:** emilin1, genetics counseling, misinformed lab reports, multiple fractures, tortuosity in multiple arteries

## Abstract

A nine-month-old male was referred with short stature, tortuosity in multiple arteries, pulmonary stenosis, and multiple fractures. Trio exome sequencing (ES) revealed a c.275_277delinsTT, p.(C92Ffs*22) *maternal variant* and a *c.1759G>T, p.(G587) paternal EMILIN1 variant. The lab report classified his biallelic EMILIN1 terminator variants as variants of unknown significance (VUSs) and did not postulate a possible relationship with a known autosomal recessive disorder. While EMILIN1 is listed in OMIM as causing an autosomal dominant disorder (OMIM # 620080), there are no current OMIM entries for clinically heterogeneous disorders with an autosomal recessive mode of inheritance variants. Importantly, at that time, biallelic loss-of-function variants in EMILIN1 had already been shown to cause an autosomal recessive disorder characterized by cutis laxa, arterial tortuosity, aneurysm formation, and bone fragility. However, the close overlap of his pleiotropic features was not acknowledged. We informed his parents that he very likely had this known condition, despite the benign lab interpretation. This scenario illustrates the potential need for clinicians and genetic counselors to carefully review and, in some cases, correct misinformed lab reports.

## Introduction

EMILIN1 protein is an elastin microfibril interfacer that is associated with connective tissue integrity, although its exact mechanism is not fully understood [1]. EMILIN1 is strongly expressed in tissues such as the connective tissue of the cardiovascular system, where it associates with elastic fibers at the interface between elastin and microfibrils [2]. The EMILIN1 gene is currently listed in OMIM as causing an autosomal dominant disorder (OMIM # 620080). However, as of June 25, 2024, there were no current OMIM entries for clinically heterogeneous disorders with autosomal recessive mode of inheritance variants. Nonetheless, on December 1, 2022, biallelic loss-of-function variants in the EMILIN1 gene were reported to cause an autosomal recessive disorder characterized by cutis laxa, arterial tortuosity, aneurysm formation, and bone fragility in six individuals [3]. We report here an infant seen in January 2024 with tortuosity in multiple arteries and recurrent fractures, almost certainly caused by the biallelic terminator variants in EMILIN1. His CLIA trio exome sequencing report, dated May 16, 2024, did not acknowledge the likely pathogenicity of these variants or the existence of either OMIM 620080 or the previous literature [3].

## Case presentation

Our proband is a nine-month-old male with short stature, tortuosity in multiple arteries, pulmonary stenosis, multiple fractures, plagiocephaly, and inverted nipples. He was born via vacuum-assisted vaginal delivery due to non-reassuring fetal heart rate tracing to a gravida 2 para 1 mother at 40 weeks and four days. Pregnancy was uncomplicated except for polyhydramnios. The postnatal course was complicated by respiratory distress due to aspiration of amniotic fluids. He was found to have multiple fractures including a clavicle, scapula, and multiple ribs after delivery. A postnatal echocardiogram revealed supravalvular pulmonary stenosis and aortic arch anomaly. An MRA showed mild tortuosity of the cervical, intracranial, and vertebral arteries, along with tortuosity of the aortic arch and great vessel origins. A renal ultrasound revealed a left duplex collecting system and mild hydronephrosis with normal renal vasculature. At two weeks of age, he had a fracture of his other clavicle. He was evaluated by orthopedics, where his fractures and X-rays were thought to be suggestive of osteogenesis imperfecta. 

He was referred to our genetics clinic for further evaluation. His family history revealed that his parents and sister were all in good health. His physical examinations were notable for short stature (height <3 percentile), plagiocephaly, and inverted nipples. His chromosomal microarray was normal. A copper level test was obtained to exclude Menkes disease, and the result was normal. DNA sequencing panels for osteogenesis imperfecta and bone fragility as well as connective tissue disorders revealed a heterozygous c.1018G>A, p.Gly340Ser paternal COL9A2 variant of unknown significance (VUS) (Table 1). A pathogenic variant in COL9A2 has been associated with epiphyseal dysplasia; however, it did not fit the patient’s phenotypic presentation of arterial tortuosity and fractures. In addition, the absence of any features suggestive of epiphyseal dysplasia in his father made this VUS less concerning. Given these inconclusive results, a trio ES was obtained, revealing a c.275_277delinsTT, p.C92Ffs22 maternal variant and a c.1759G>T, p.G587 paternal EMILIN1 VUS. However, the lab report from May 2024 stated that no diseases currently described were associated with EMILIN1 variants (Table 2).

**Table 1:** DNA sequencing panels for osteogenesis imperfecta and bone fragility as well as connective tissue disorders result DNA sequencing panels for osteogenesis imperfecta and bone fragility as well as connective tissue disorders revealed a heterozygous c.1018G>A, p.Gly340Ser paternal COL9A2 VUS. VUS: variant of unknown significance

Gene	Disease	Mode of inheritance	Variant	Zygosity	Inherited from	Classification
COL9A2	Epiphyseal dysplasia	Autosomal dominant	c.1018G>A, p.Gly340Ser	Heterozygous	Father	VUS

**Table 2:** Trio ES result Trio ES revealed a c.275_277delinsTT, p.C92Ffs*22 maternal and a c.1759 G>T, p.G587* paternal EMILIN1 VUS. ES: exome sequencing; VUS: variant of unknown significance

Gene	Disease	Mode of inheritance	Variant	Zygosity	Inherited from	Classification
EMILIN1	None currently described	Unknown	c.275_277delinsTT, p.C92Ffs*22	Heterozygous	Mother	VUS
EMILIN1	None currently described	Unknown	c.1759 G>T, p.G587*	Heterozygous	Father	VUS

## Discussion

Here we describe a proband with short stature, tortuosity in multiple arteries, pulmonary stenosis, multiple fractures, plagiocephaly, and inverted nipples with biallelic EMILIN1* *terminator variants referred to as VUS detected in his trio ES. 

EMLIN1 gene is known to play a role in nervous system development, the functioning of vascular elastic fibers, and skin homeostasis [2,4]. Biallelic loss-of-function variants in the EMLIN1 were reported in six affected individuals from four families with aortic tortuosity [3]. Other reported clinical phenotypes include tortuosity of other arteries, aortic root dilation, recurrent fractures, hydronephrosis, thin velvety skin, cutis laxa, joint hyperlaxity, respiratory distress, increased arm span, seizures, hypotonia, congenital heart disease, intrauterine growth restriction, and distinctive craniofacial features (Table 3) [3]. 

**Table 3:** Comparison of clinical features of individuals with biallelic loss-of-function variants in EMLIN1 in Adamo et al. [3] and our proband Our proband’s phenotypic presentations overlap well with the clinical features of individuals reported in Adamo et al. [3].

Reported features (Adamo et al., 2022) [3]	Our proband
Arterial tortuosity, tortuosity of other arteries, aortic root dilation, congenital heart disease	+
Recurrent fractures	+
Hydronephrosis	+
Thin velvety skin, cutis laxa, joint hyperlaxity, increased arm span	-
Respiratory distress	+
Seizures	-
Hypotonia	-
Intrauterine growth restriction	-
Distinctive craniofacial features	-

Our proband’s main phenotypic presentations of tortuosity in multiple arteries and recurrent fractures and terminator variants overlap well with the phenotypes of multiple individuals reported to result from biallelic loss-of-function variants in the EMILIN1 (Table 3) [3]. Nevertheless, neither the publication nor the close overlap of his pleiotropic features with it were included in his lab report. Thus, neither the concern over the possible pathogenicity of his variants nor the possibility that his very unusual phenotypic features meshed well with those seen in others with biallelic loss-of-function EMILIN1 variants were reported. By comparing his presentation with currently available information found through simple Google searches using 'EMILIN1 and tortuosity' or 'EMILIN1 and fractures' as search terms, we concluded that his two variants were likely pathogenic and meshed well with a previously reported disorder caused by biallelic terminator variants in EMILIN1.

Our proband's findings and our different conclusions from his trio ES report raised several ethical questions. First, his lab report did not acknowledge the possible pathogenicity of his biallelic terminator variants. Second, it did not acknowledge previous literature regarding his likely disease [3]. Third, we believed that several ethical issues warranted challenging his lab report. These included the following: a) regarding beneficence, we concluded that revealing his diagnosis could be beneficial to his family and healthcare providers, b) regarding nonmaleficence, we thought that failing to reveal our suspected diagnosis could cause harm, and c) regarding truth-telling, the benefits of being truthful included avoiding possible harm from multiple tests and a prolonged medical odyssey resulting from the lab oversights. Therefore, we chose to inform his parents that we believed he very likely had this known genetic disorder, which could recur in 25% of their future pregnancies, and provided them with details about the condition, despite the benign lab interpretation. This scenario illustrates the potential need for clinicians and genetic counselors to discuss the limitations of ES and other genetic tests when obtaining informed consent and returning results. They should carefully review the results and, if clinically indicated, consider correcting misinformed lab reports [5]. It also illustrates the limitations of timely curation of genetic databases when only a single publication on the disease exists, due to its rarity and/or the requirement for unusual testing, such as MRA, and highlights the need for simple online searches to ensure that pertinent but non-curated publications are identified.

## Conclusions

We report a case of tortuosity in multiple arteries with multiple fractures, likely caused by biallelic terminator variants in EMILIN1, whose trio ES report did not acknowledge the likely pathogenicity of the alleles, nor the current literature regarding his likely resulting disease. We informed his parents that he very likely had this known condition and provided details about the condition, despite the benign lab interpretation. This scenario illustrates the potential need for clinicians and genetic counselors to carefully review, and in some cases, correct misinformed lab reports.
